# Nanoparticle mediated drug delivery of rolipram to tyrosine kinase B positive cells in the inner ear with targeting peptides and agonistic antibodies

**DOI:** 10.3389/fnagi.2015.00071

**Published:** 2015-05-19

**Authors:** Rudolf Glueckert, Christian O. Pritz, Soumen Roy, Jozsef Dudas, Anneliese Schrott-Fischer

**Affiliations:** ^1^Department of Otolaryngology, Medical University of InnsbruckInnsbruck, Austria; ^2^University Clinics of Innsbruck, Tiroler Landeskrankenanstalten GmbH-TILAKInnsbruck, Austria; ^3^Department of Genetics, Institute of Life Sciences, Hebrew University of JerusalemJerusalem, Israel

**Keywords:** inner ear, drug delivery, BDNF-TrkB signaling, rolipram, lipid core nanocapsules, polymerosom nanoparticles, quantum dot nano-suspensions, silica nanoparticle, explant culture

## Abstract

**Aim:** Systemic pharmacotherapies have limitation due to blood-labyrinth barrier, so local delivery via the round window membrane opens a path for effective treatment. Multifunctional nanoparticle (NP)-mediated cell specific drug delivery may enhance efficacy and reduce side effects. Different NPs with ligands to target TrkB receptor were tested. Distribution, uptake mechanisms, trafficking, and bioefficacy of drug release of rolipram loaded NPs were evaluated.

**Methods:** We tested lipid based nanocapsules (LNCs), Quantum Dot, silica NPs with surface modification by peptides mimicking TrkB or TrkB activating antibodies. Bioefficacy of drug release was tested with rolipram loaded LNCs to prevent cisplatin-induced apoptosis. We established different cell culture models with SH-SY-5Y and inner ear derived cell lines and used neonatal and adult mouse explants. Uptake and trafficking was evaluated with FACS and confocal as well as transmission electron microscopy.

**Results:** Plain NPs show some selectivity in uptake related to the *in vitro* system properties, carrier material, and NP size. Some peptide ligands provide enhanced targeted uptake to neuronal cells but failed to show this in cell cultures. Agonistic antibodies linked to silica NPs showed TrkB activation and enhanced binding to inner ear derived cells. Rolipram loaded LNCs proved as effective carriers to prevent cisplatin-induced apoptosis.

**Discussion:** Most NPs with targeting ligands showed limited effects to enhance uptake. NP aggregation and unspecific binding may change uptake mechanisms and impair endocytosis by an overload of NPs. This may affect survival signaling. NPs with antibodies activate survival signaling and show effective binding to TrkB positive cells but needs further optimization for specific internalization. Bioefficiacy of rolipram release confirms LNCs as encouraging vectors for drug delivery of lipophilic agents to the inner ear with ideal release characteristics independent of endocytosis.

## Introduction

### Background

The problem of hearing impairment is growing. British MRC Institute of Hearing Research (www.hear-it.org, 2011) estimates that the total number of people suffering from hearing loss of more than 25 dB will exceed 700 million by 2015. Systemic pharmacological intervention to treat sensorineural hearing loss suffers to show significant effects. Due to the demographic development in all western countries age related hearing loss can be expected to increase continuously over the next decades. Effective pharmaceutical treatments are rare.

The isolated anatomical position, a blood-labyrinth barrier and low blood flow may be reasons that drugs do not reach therapeutic levels in the inner ear. Local inner ear treatment via the round window membrane (RWM) seems to be an effective natural pathway for drug application. Intratympanic administration of insulin like growth factor 1 (IGF-1) (Nakagawa et al., 2014) to treat sudden sensorineural hearing loss shows some promising results, studies of glucosteroids applied that way differ in their significance (Spear and Schwartz, 2011; Ng et al., 2014). New strategies for preserving low frequency hearing after cochlear implantation raise growing needs to protect sensorineural cells from trauma with effective treatments. There are candidate drugs that show efficacy to restore damage of the sensory receptor and spiral ganglion neurons (SGNs). Inhibition of caspases prevents or delays hair cell death and may preserve hearing/balance function (Wang et al., 2003; Cheng et al., 2005; Dinh and Van De Water, 2009) and neurotrophic factors have been shown to restore synaptic connection after noise trauma and promote neuron outgrowth after deafferenciation (Glueckert et al., 2008) as well as neural survival (Pettingill et al., 2011). Inhibition or scavenge of reactive oxygen species is another strategy to meliorate cell damage (Henderson et al., 2006; Yamasoba et al., 2013). Another putative therapeutic to provide anti-apoptotic signaling is rolipram, a posphodiesterase 4 inhibitor which affects neuronal survival via proteinkinase-A-mediated increase in cAMP responsive element binding protein activity and the expression of downstream targets as brain-derived neurotrophic factor (BDNF) and its high affinity receptor tropomyosin receptor kinase B (TrkB) (Asanuma et al., 1996; Nibuya et al., 1996).

### Nanoparticle mediated drug delivery (NPDD)

Nanoparticles (NPs) as vehicles to reach target cells in the inner ear may overcome limitations of pharmaceutical formulations and specifically target certain cell types. Some drugs show severe side effects (Suckfuell et al., 2007; Weissmiller and Wu, 2012), solubility, and stability may be poor (Gupta and Dixit, 2011), here NPs may act as a versatile tool to avoid off target effects on healthy tissue. Several NPs have been found to cross the RWM barrier (Moss and Wong, 2006; Roy et al., 2012; Liu et al., 2013), this is a prerequisite for atraumatic penetration of the inner ear. Fluid pathways in the cochlea (Rask-Andersen et al., 2006; Salt and Plontke, 2009) and *in vivo* experiments with NPs showed the feasibility to reach target structures via this route such as the sensory epithelium and SGNs (Tamura et al., 2005; Buckiova et al., 2012). Passive diffusion as well as magnetic force enhancement for paramagnetic NPs was reported to reach at least the basal portion of the cochlea (Tamura et al., 2005; Ge et al., 2007; Du et al., 2013). Cell-NP interactions largely depend on particles' physicochemical properties including surface charge, size, shape as well as surface chemistry that builds up the protein corona with body fluids under *in vivo* conditions (Shang et al., 2014) and adds new biological properties. Multivalent attachment of small molecules or antibodies adsorbed to the NP surface that interact with membrane associated proteins may activate cell's uptake machinery to internalize the particles. Cell specific internalization with drug bioefficacy and biosafety of the nanocarrier is the final aim. Within a European Union Consortium called “NanoEar” (contract nr. NMP-20043-.4.1.51-1) several NPs were developed to selectively target sensorineural structures within the cochlea as vehicles for future pharmacotherapies. Some results are presented here.

### TrkB as target for NPDD

In the inner ear SGNs are an indispensable element for the signal transduction from the hair cell to the brain (Bibel and Barde, 2000; Rubel and Fritzsch, 2002). In pathologic conditions, these cells are prone to cell death. For that reason, the preservation of those cells is paramount and renders these cells a target for NPDD. There is a neurotrophic relationship between hair cells and supporting cells, both providing neurotrophins, and SGNs, receiving the neutrophins (Zilberstein et al., 2012). Supplementation of BDNF and neurotrophin 3 (NT-3) after hair cell loss and subsequent damage to the supporting cells leads to a higher survival rate of SGNs (Deng et al., 2004; McGuinness and Shepherd, 2005; Wang and Green, 2011). Especially the TrkB is of particular interest because as alternative to BDNF, there is a number of agonistic molecules including antibodies (Cazorla et al., 2011) that circumvent the low stability of the BDNF protein. Since TrkB is expressed in adult human SGNs (Liu et al., 2011) and adult as well as developing mice inner ears (Bitsche et al., 2011), TrkB is an ideal target for NPDD targeting the SGNs. On the one hand, TrkB can act as label for SGNs to mediate specific binding and endocytosis of the NPDD. On the other hand, TrkB itself can be activated by an agonistic surface modification and thus contribute to mitogen-activated protein kinase (MAPK), AKT and phospholipase C γ (PLCγ)-mediated neuronal survival signaling (Klein et al., 1989, 1993; Minichiello et al., 1998; Atwal et al., 2000; Watson et al., 2001; Mizoguchi and Nabekura, 2003; Gruart et al., 2007). In parallel the NPDD is still capable of delivering an anti-apoptotic drug such as rolipram (Meyer et al., 2012). Co-application of BDNF and rolipram strongly enhances the survival promoting effect of BDNF (Kranz et al., 2014). BDNF and rolipram may also stimulate the pro-apoptotic low affinity p75 receptor in parallel, so excessive stimulation needs to be prevented, as too much of pro survival signals may lead to tumorgenesis (Geiger and Peeper, 2007).

### Targeting ligands

Conjugated targeting ligands and other surface modifications on NPs serve various purposes like to mediate the permeation through epithelia, to activate signaling cascades, and to mediate the specific uptake in distinct cell populations. Ligands include small molecules, peptides, protein domains, antibodies, and nucleic acid aptamers, with all classes having unique attributes reviewed previously (Friedman et al., 2013). Conjugating targeting ligands such as in terms of surface modifications, antibodies have already demonstrated in several fields to function when surface-grafted (Beduneau et al., 2008; Ulbrich et al., 2009; Choi et al., 2011; Fiandra et al., 2013). For extracellular TrkB activation, the agonistic anti-TrkB antibody from clone 6B10 (NB110-94149) Novus Biologicals antiTrkB mouse monoclonal (at 1 mg.ml^−1^ corresponds to 6.84 μM) was used to test TrkB activation in SH-SY5Y-G7 cells (passage 3). The agonistic anti-TrkB antibody 29D7 (Wyeth Research, Pfizer, Connecticut, USA) has already been shown to bind and activate TrkB when surface grafted to iron NPs (Steketee et al., 2011). Therefore, the use of 29D7 might be an appropriate means to establish an NPDD specifically targeting and activating SGN membrane resident TrkB. Here 29D7 monoclonal TrkB antibody-surface grafted 50 nm silica NPs (aTrkB NP) were used to assess the functionality of the antibody surface-modification to bind and activate TrkB in the HEI-OC1 and VOT-N33 cell culture model. The binding of TrkB by the immuno-NP in the cochlea and the localization after application was further tested in cochlea whole-organ-culture. Much smaller than antibodies short homing peptides offer several advantages such as high packing density, less immunogenicity and often higher purity, though design can be challenging. Typically these peptides are identified via the screening tool phage display (Kehoe and Kay, 2005; Deutscher, 2010), allowing selection of peptide sequences with increased affinities to a specific target of choice displayed on bacteriophage capsids that are collected and amplified with infected *E. coli*. Numerous sequences have been tested (Friedman et al., 2013), we designed peptides based on Ma et al. (2003) and summarized here (Ranjan et al., 2012).

## Material and methods

### Nanocarriers and ligands

Polymerosomes, lipid core nanocapsules (LNCs), silica as well as quantum dot NPs (QDotNPs) were used for the presented study. Peptide ligands are summarized in **Figure 5** and described previously (Ranjan et al., 2012). 29D7 monoclonal anti-TrkB-antibody was kindly provided by Wyeth Research, Pfizer®, Connecticut, USA and characterized previously (Steketee et al., 2011).

**Polymerosomes**: Amphiphilic block copolymers can self-assemble into polymersome NPs in an aqueous environment. Polymersomes are vesicular, nano-sized spheres that encapsulate an aqueous solution. The synthetic amphiphilic block copolymers consist of hydrophilic and hydrophobic units joined together. Poly lactic/glycolic acid is a charged biodegradable copolymer composed of lactic and glycolic acid monomers and forms here Polyethylene glycol-block-polycaprolactone (PEG-b-PCL) NPs. NP manufacturing and ligand ligation was described previously (Roy et al., 2010; Pritz et al., 2013b).

**Lipid Core Nanocapsule (LNC)** structure resembles that of lipoproteins with a lipidic core (triglycerides, mineral oils) surrounded by a amphiphilic shell formed by lecithin and stearate of Polyethlenglycol (PEG), where lecithin is located in the inner part of the shell. NPs synthesis and characterization is described here (Bastiat et al., 2013) in detail. Liposomes half-life can be extended by PEG-derivatized lipids (Uster et al., 1996).

Mesoporous **silica NPs** display high specific pore volume and surface area for high drug loadings. Particle size can be controlled more precisely and functionalization with different surface modification and tags make them ideal for imaging purposes. NPs were 50 nm in diameter and manufactured by MicroMod®, Rostock Germany and described previously (Pritz et al., 2013a), FITC fluorochrome served as visualization agent. 29D7 monoclonal anti-TrkB-antibody (Wyeth Research, Pfizer) surface-grafted silica NPs (42-36-501; S08412, MicroMod®) and bovine serum albumin (BSA) surface-grafted silica NPs (BSA-NP) (42-21-501; S08912, MicroMod®) were tested.

**Quantum Dot NPs** are made from emulsions, containing iron oxide, and quantum dots, they are composite NPs composed of two block copolymers, PLLA-mPEG: Poly(L-lactic acid)-block-poly(ethylene glycol). We investigated the internalization efficiency of these particles in neuronal-like cell cultures and cochlear explants. These NPs were manufactured by the Division of Functional Materials at the Royal Institute of Technology, Stockholm, Sweden, are biodegradable and resorbable with a hydrodynamic diameter of 423 (± 8) nm for orange QDotNPs and 200 (± 15) nm for green QDotNPs. CdO powder (Fluka) is added to a mixed solvent system containing 1-octadecene (Aldrich) and oleic acid (Sigma Aldrich), and heated at 160°C under nitrogen for 1 h, to obtain cadmium oleate. This is stored under nitrogen, and is usable for 2 weeks. Se powder is added to a mixed solvent system containing TOP (Trioctyl-phosphine, Fluka) and 1-octadecene and heated at 250°C under nitrogen for 1 h, to obtain trioctylphosphine selenide (TOPSe, Aldrich). This is stored under nitrogen and is usable for 2 weeks. In a typical synthesis, cadmium oleate is added to 1-octadecene and oleylamine (Fluka) in a 3-necked flask and the mixture is heated to 250°C under nitrogen. At this temperature, TOPSe is injected into this mixture. Depending on the desired size of the QDots, this reaction is stopped at variable times (typically ranging from 20 to 1800 s), by injecting excessive amount of solvent at room temperature. QDots are a very stable fluorochrome and ideal for imaging purposes. Because of their extremely small size and optical resolution, they are also well suited for tracking the molecular dynamics of intracellular and/or intercellular molecular processes over long time scales (Pathak et al., 2006). Orange Quantum dots: CdSe/CdS quantum dots (with orange emission) were payloaded within the NPs, where PLGA: poly(lactic-co-glycolide) is used as a matrix material and carboxylic group as a surface. Green Quantum dots: CdSe/CdS quantum dots (with green emission) were payloaded within the NPs, where PLLA-mPEG: poly(L-lactic acid)-block-poly(ethylene glycol) is used as a matrix material and PEG as a surface. Characterization of CdSe quantum dots: A sample of NP suspension is characterized by ultraviolet and visible spectroscopy, and photoluminescence spectroscopy by depositing 1 ml of suspension in a polymethyl methacrylate cuvette at room temperature. Transmission electron microscope (TEM) analysis was performed after depositing one drop of NP suspension on a copper grid coated with formvar and carbon and letting to dry.

### Cell culture

**HEI-OC1 cells** were isolated by Dr. Federico Kalinec (Yorgason et al., 2010) and are used as a model for TrkB positive cells in the inner ear, when cultured under 33°C and 10% CO_2_. Furthermore, after differentiation at 39°C and 5% CO_2_ these cells represent a model for inner and outer hair cells of the mouse cochlea. Cells were grown as described previously (Pritz et al., 2013a). For size dependent uptake cells were plated on 21 × 26 mm cover slips. The cover slips were placed into 6-well plates; cells were plated at 10^4^/ml. NP treatment occurred usually 24 h after plating. NPs were used in 500-, 1500- and 15,000- fold final dilution. For antibody coated silica NPs cells were plated 12 h before the start of the experiment. Cells were incubated with 9.5 × 10^9^ NP.ml^−1^ 29D7 monoclonal TrkB-antibody (Wyeth Research, Pfizer) surface-grafted silica NPs (42-36-501 S08412, MicroMod®, Rostock Germany) and BSA surface-grafted silica NPs (42-21-501 S08912, MicroMod®). Each particle type was tested in two different conditions creating a competitive assay: binding and blocking conditions. Binding conditions: Cells were incubated with NPs at pH 7.4 in serum-free conditions in Dulbecco's Modified Eagle (DMEM) low-glucose Medium (E15-005 PAA Pasching, Austria) for 90 min. Blocking conditions: Cells were incubated with NPs at pH7.4 together with free 29D7 monoclonal TrkB-antibody (Pfizer®, USA) in 1 nM concentration (63-fold excess) for 90 min. The experiment was carried out at 4°C to prevent endocytosis and at 33°C the culturing temperature of the cells to permit endocytosis. According experiments were performed with BSA-grafted silica-NPs under same conditions. In each group 15–20 cells were analyzed.

The auditory neuroblast cell line **US/VOT-N33 (N33)**, which is conditionally immortal, was kindly provided by Matthew Holley and shall serve as an *in vitro* model for SGNs. Culture conditions were described previously (Nicholl et al., 2005). In brief, cells are grown in minimum essential medium (MEM) containing 10% fetal calf serum at 33°C, 10% CO_2_ and after 3 h serum deprivation used for experiments. Antibody coated silica NPs were applied like with HEI-OC1 cells.

The **neuroblastoma cell line SH-SY5Y** was differentiated by all-trans-retinoic acid (ATRA) treatment that induces expression of TrkB, but not of TrkA receptor, and mediates biological responsiveness to receptors for the neurotrophins BDNF and NT-4/5 as described previously by our group (Ranjan et al., 2012). As an alternative to SH-SY5Y cells, **stable TrkB-transfected G7** cells were received from Garrett Brodeur, M.D. (Children's Hospital of Philadelphia, Philadelphia, Pennsylvania, USA) and maintained and cultured as referenced (Eggert et al., 2002). TrkB overexpressing SH-SY5Y cells were grown in the presence of the selection antibiotic as described elsewhere (Soumen et al., 2012). Cells were plated 12 h before the start of the experiment. Cells were serum starved for 3 h. Then cells were incubated with 5.5 nM BDNF, 1.9 × 10^10^ NP.ml^−1^ immuno-NP, 1.9 × 10^10^ NP.ml^−1^ immuno-NP and 9.5 × 10^9^ NP.ml^−1^ BSA-NP for 90 min. Cells were rinsed three times in Tris-buffered saline (TBS) buffer and subsequently harvested with a cell scraper and lysed in lysis buffer (1% v/v NP-40, 50 mM Tris pH 8, 150 mM NaCl, 5 mM ethylenediaminetetraacetic acid (EDTA)). Protein quantification was subsequently performed by a Bradford assay (500-0001, BioRad, Vienna, Austria), at 595 nm using a BioPhotometer Plus (Eppendorf® Germany, Wien, Austria).

### Western blotting

Fifteen microgram of protein lysate was loaded in each slot. SDS-PAGE was carried out at 20 mA for 1 h in a 12% bisacrylamid gel using a Mighty Small II Deluxe Mini Vertical Electrophoresis Unit (SE260, Hoefer, Holliston, USA). Protein was subsequently transferred to a PVDF membrane (88518, Thermo Scientific®, Waltham USA) using a Maxi-BlotTank fully wet blotter (340.000, GP Kunststofftechnik, Germany) at 60 mA for 4 h at 4°C. Membranes were subsequently stained with anti-phospho-Erk1/2 antibody (#9101 Cell Signaling Technologies) in 1:1000 dilution and goat anti rabbit horseradish peroxidase (HRP) conjugated (31460, Thermo Scientific®) in 1:10 K dilution as described in detail in Wiedemann et al. (2006). Signal detection was performed by enhanced chemi-luminescence using SuperSignal West Dura Chemilumenescent Substrate (37071, Thermo Scientific®) and CL-Xposure X-Ray films (34091, Thermo Scientific®). X-Ray films were subsequently scanned.

### Organotypic culture and zero-gravity whole organ culture of the cochlea

Organotypic mouse culture was performed on neonatal P1–P3 mouse cochleae as described previously (Roy et al., 2010). Zero-gravity whole organ culture of adult cochlea was done with P18–P30 mice anesthetized by intraperitoneal injection of ketanmin hydrochlroid (84 μg.g^−1^ bodyweight, Ketasol® Graeub Veterinary Products, Bern Switzerland), 0.25 μg.g^−1^ bodyweight atropine sulfate (Atropium Sulfaticum, Nycomed® Austria GmbH, Linz, Austria), and 6.7 mg.g^−1^ bodyweight xylazin (Bayer® Healthcare, Berlin, Germany) in physiologic saline and subsequently killed by cervical dislocation. The cochleae were removed from the scull. All animal experiments were approved by the Austrian Ministry of Science and Research and conformed to the Austrian guidelines on animal welfare and experimentation (BMWF-66011/0109-II/3b/2012). Stapes, RWM as well as the bone covering the apical portion of the cochlea and 270° along the basal turn covering the scala tympani was removed. The dissected cochleae were then transferred to culturing medium pre-warmed to 37°C. The culturing medium was composed of 100 U.ml^−1^ penicillin (P7794-10MU, Sigma Aldrich, Vienna, Austria), B27 supplement (17504044, Invitrogen, Lofer, Austria), 5 mM L-glutamine (BE 17-505E, Lonza), Neurobasalmedium (21103-049, Invitrogen) at pH 7.4. The culturing was performed in a rotary cell culture system (RCCS-4SC, Synthecon® Incorporated, Housten, USA) in 10 ml disposable HARV-10 vessels (Synthecon® Incorporated) for 2 h. The culturing medium was then supplemented with 9.5 × 10^9^ aTrkB NPs for immuno-conjugate NPs for 24 h or LNC particles loaded with rolipram as described by Meyer et al. (2012). LNCs were provided in a concentration of 4 × 10^15^ particles/ml with a hydrodynamic diameter of 52 ± 5 nm. 1:100 and 1:1000 dilution of LNC particles loaded with 2 μM rolipram with adding 50 μM cisplatin to the culture medium were tested in comparison with 50 μM cisplatin and culture medium alone for 24 and 48 h.

### Processing of cultured cochleae

After culturing, cochleae were washed in phosphate buffered saline (PBS) and subsequently fixed in 2% paraformaldehyde in PBS overnight at 4°C. The specimens were decalcified using 20% EDTA in PBS for 4 h at 37°C. Specimens were then infiltrated by in an ascending series of 10 and 15% D-sucrose in PBS, and finally a mixture of 15% D-sucrose and 50% OCT-compound (4583, Tissue-Tek®, Finetek Sakura® Europe, Leiden, Netherlands). Final infiltration was performed in 100% OCT®-Compound overnight. Cochleae were rapidly frozen in OCT®-compound at –78°C as in a 1 + 1 EtOH:dry ice mixture. One half of the frozen cochleae were subsequently cryo-sectioned in 10 μm sections. The other half of the cochleae were thawed and post fixed in 2% glutaraldehyde in PBS at pH 7.4. Cochleae were than incubated in 1% OsO_4_ for 1 h at 4°C in 0.05 M cacodylate buffer. Specimen were then dehydrated in an ascending EtOH series and embedded in Epon 812 (Electron Microscopy Science, Hatfield, UK, 14120) as described elsewhere (Thaler et al., 2011; Pritz et al., 2013a).

### Immunostaining

Cell culture: early endosome antigen (EEA1) and lysosomal-associated membrane protein (LAMP-1) staining was performed as described previously (Pritz et al., 2013a). Rotary culture sections: After 3 × 5 min in PBS blocking and permeabilization (1 h 1% BSA and 5% normal goat serum in PBS + 0.05% Tween 20 at 4°C) primary antibodies rabbit-anti cleaved caspase-3 (CC3) 1:400, (Asp175, Cell signaling 9661L) Abcam® rabbit polyclonal anti-beta-III-Tubulin ab 1:200 or rabbit IgG isotype control was applied for 1 h at 4°C. After thorough wash in PBS, 2nd antibody Alexa® Fluor 647 F(ab')2 fragment goat anti rabbit (111-606-047, Jackson Immunoresearch®, PA, USA) 1:1500 in 0.5% (w/v) BSA was incubated overnight at 4°C in dark and subsequently 30 min 37°C and mounted with Vectashield® including DAPI (Vector Laboratories® Ltd, UK). Phalloidin FITC (Sigma® P5282; 1:80) was applied according to the manufacturer's protocol. Control samples were substituted with isotype matching rabbit immunoglobulins (rabbit polyclonal IgG Abcam®, ab27478, 1:200). These controls were consistently negative.

### Confocal microscopy

NP treated cells were illuminated by a 488 nm laser, at pinhole = 3 AU, resulting fluorescence was collected between 505 and 545 nm. Cochlea sections and NP treated cells were examined using Zeiss® LSM510 Meta (Zeiss®, Oberkochen Germany) using ZEN 2009. FITC was excited by 488 nm laser. For pairwise comparison of NP binding cells were imaged with a pinhole of 3 AU, illumination by a 488 nm laser, resulting fluorescence was collected from 505 to 545 nm. Due to the high autofluorescence in adult cochlea explant sections, the FITC in NPs was detected using lambda stacks from 490 to 700 nm. To distinguish FITC fluorescence from autofluorescence ratiometric detetection was performed. Fluorescence at 520 nm was divided by 565 nm. Alexa 647 was excited with a 633 nm Laser and detected with 650 bandpass filter, DAPI excited with 405 nm laser as described previously (Glueckert et al., 2008).

### Transmission electron microscopy

For electron microscopy, epon blocks were sectioned to 80 nm sections. Electron microscopy was performed using a Zeiss® Libra 120 EFTEM (ZEISS, Oberkochen, Germany) as described in Pritz et al. (2013a) and Thaler et al. (2011).

### Flow cytometry

SHSY5Y cells were grown in DMEM/F12 1:1 medium (PAA, Linz, Austria) supplemented with 2 mM L-glutamine, penicillin (20 units/ml), streptomycin (20 mg/ml), and 15% (v/v) heat-inactivated fetal calf serum (PAA®) (Ranjan et al., 2012). Cells were maintained at 37°C in a saturated humidity atmosphere containing 95% air and 5% CO_2_. Cells were seeded at an initial density of 10^4^ cells/cm^2^ in culture dishes (Unilab®, Innsbruck, Austria). All-trans-RA (Sigma®, Darmstadt, Germany) was added on the day of plating at a final concentration of 10 μM in DMEM/F12 with 15% fetal calf serum, and after that, daily. After 5 days in the presence of ATRA, cells were treated with LNC nanoparticles at 50 μg/ml final concentration for 5 or for 24 h, and analyzed by flow cytometry. The prepared LNCs contained Nile Red, which was used as a model of amphiphilic carried drug, and was also detectable by the excitation of the 488 nm laser source in flow cytometry. The particles were prepared without peptide conjugate and with A415, A747, scrambled Scr-A415, and scrambled Scr-A747 peptides, where A415 and A747 were intended as TrkB-targeting peptides, the other two were synthesized with scrambled sequences. The hydrodynamic diameter of the particles ranged 49–58 nm (**Figure 5**). LNCs labeled with Nile Red were intact particles in flow cytometer within the 24 h of experimentation. After the desired incubation period, cells were removed and collected from the culture dishes by trypsinization (PAA®) washed once with serum-containing culture medium and resuspended with Isoton II sheath (Beckman Coulter®, Brea CA, USA) at 10^6^ cells/ml sheath. Cell suspension was observed using a Coulter® XL-MCL flow cytometer (Beckman Coulter®) using the FL-2 channel with excitation of 488 nm and detection of the emission at 560–590 nm. These conditions are optimal for detecting Nile Red in lipid environment, as expected in LNCs (Greenspan and Fowler, 1985). Not stained cells were used as control and showed 0.9–2.1% Nile Red-like signal. These events were used for gating cells in the forward-side scatter scattergrams. The forward-side scatter scattergrams of LNC-incubated cells were comparable with the controls. The FL-2 signal of the LNC-treated cells has shown an increase. Percentage of cells showing increased signal (above the control level) was determined by the software Expo 32 (Coulter®), and was considered as Nile red positive cells.

### Statistical analysis

Experiments were planned as pair wise comparisons between binding and blocking conditions. When normal distribution was detected using Kolmogorov-Smirnov test, unpaired *t*-test was used at an alpha of 5%. When data was not normally distributed and transformation with monotonous functions failed to transform to normal distribution non-parametric tests were used.

## Results

### NP uptake in cochlear tissue

**Figure 1:**
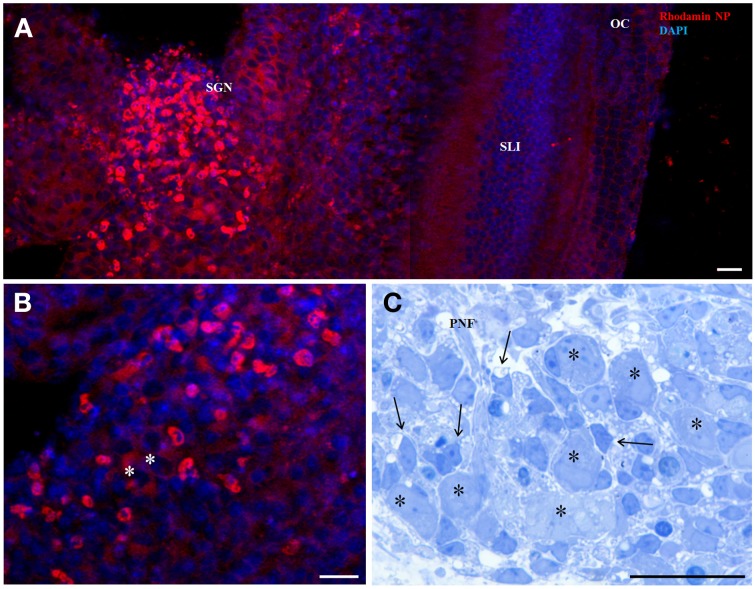
**50 nm Rhodamin labeled LNC NPs without any surface functionalization after 24 h incubation in an organotypic cochlear culture (**A,B** confocal projection)**. Panel **(A)** shows higher uptake in in the region of the spiral ganglion neurons (SGN). Panel **(B)** is a magnified view of the SGN region and identifies small cells surrounding the neurons (asterisks) with intense staining. Panel **(C)** corresponds to this region in a semithin section with big type I neurons (asterisks) and satellite glia cells (arrows) that establish the glia sheath of neuron somata. SLI, spiral ligament; OC, organ of Corti; PNF, peripheral nerve fibers. Scale bars **(A)** 50 μm; **(B)** 20 μm; **(C)** 20 μm.

The interface of LNCs, a highly packed monolayer of surfactants, has been modified by the post-insertion process carried out with pegylated amphiphilic phospholipids. This assay allowed us to introduce reactive amino groups on the surface, easily grafted with Rhodamine B molecules in the matrix material and was applied at a final concentration of 6.45 × 10^14^ particles/ml. Figures 1A,B shows the concentration gradient in different cell types in an organotypic culture of a mouse inner ear 3 days postnatal (P3). Penetration into the cytoplasm was observed all over the explant but enhanced staining was seen in the region of the SGNs, no signal was detected in any cell nuclei. Not the big bipolar neurons but smaller surrounding cells show highest uptake corresponding to satellite glia cells (SGC) that migrate from the neural crest to ensheath all neurons at a later stage of inner ear development. Figure 1C illustrates the anatomy of SGNs in a plastic embedded tissue with incomplete sheath of SGC. This depicts some selective nature of these LNC particles to show a preference for a certain cell type at a certain developmental stage.

### Size-dependent internalization

**Figure 2:**
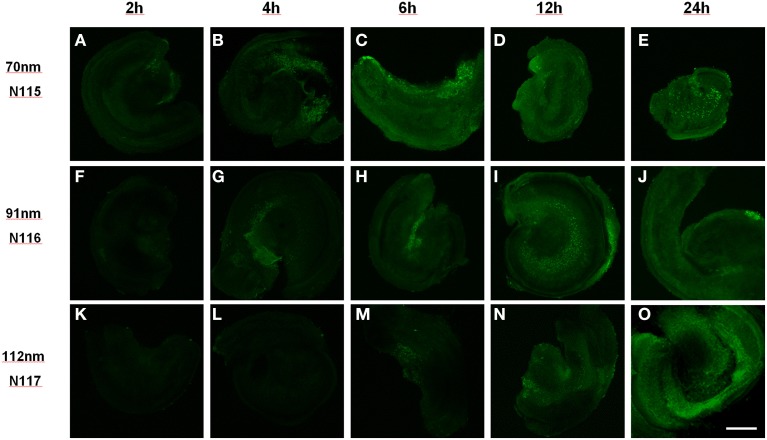
**Size dependent uptake of polymerosom NPs of different hydrodynamic diameter (N115 = 70 nm, N116 = 91 nm, N117 = 112 nm) in P3 mouse organotypic culture from 2 to 24 h of incubation: smallest particles show uptake within 2 h while biggest need 6 h**. Scale bar 200 μm, **(A–E)** 70 nm, **(F–J)** 91 nm, **(K–O)** 112 nm particles exposed for 2 h **(A,F,K)**, 4 h **(B,G,L)**, 6 h **(C,H,M)**, 12 h **(D,I,N)** and 24 h **(E,J,O)**.

NP size has a big influence on uptake kinetics and cell type selectivity. We tested different polymerosom NPs with zeta ξ potential = 1 mV and different mean hydrodynamic diameter (MHD) such as N115 (MHD = 70.2 nm), N116 (MHD = 91.2 nm) and N117 (MHD = 111.6 nm) in a concentration range in between 0.05 and 0.25 mg/ml with the different incubation time like as 2, 4, 6, 12 and 24 h in organotypic cultures of P1–P3 mice as described previously (Roy et al., 2010). Uptake of NPs was seen in an increasing time dependent manner from 2 to 24 h (Figure 2).

**Figure 3:**
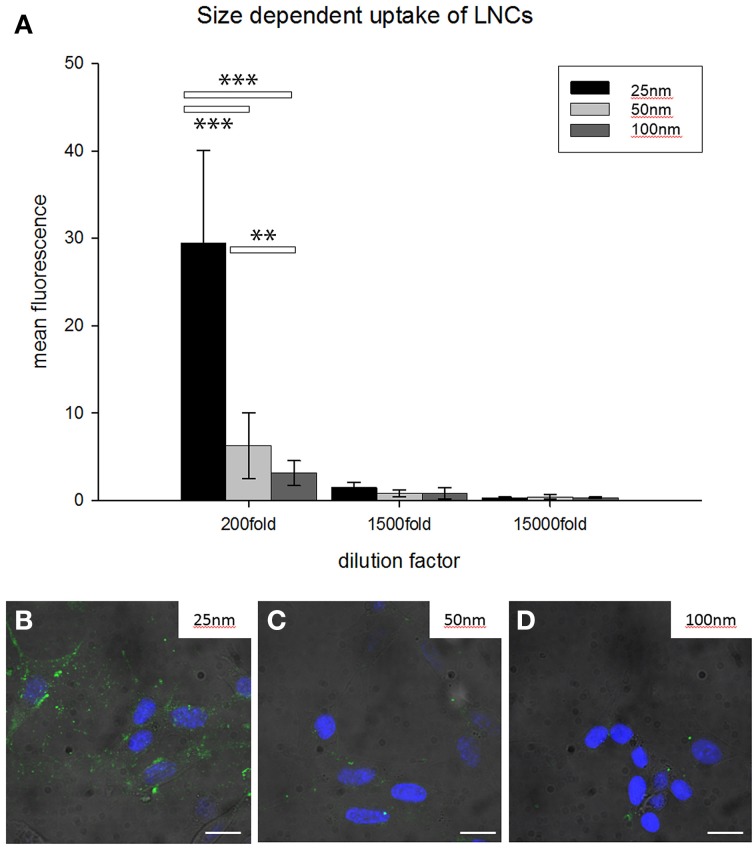
**Size-dependent internalization of unfunctionalized FITC-loaded LNCs in HEI-OC1 cells**. **(A)** Bar chart classifies 25 nm-sized NPs to be internalized most efficiently while 100 nm LNCs were internalized with the least efficiency (^**^p < 0.01, ^***^p < 0.001, ANOVA, error bars denote standard deviation). 200–1500 times dilution of LNCs shows significant differences. **(B–D)** Representative examples of HEI-OC1 cells treated with FITC-loaded LNCs (200 times dilution); diameter of LNCs is denoted in the upper right of the micrograph. Green fluorescence is FITC from LNCs, blue fluorescence is DAPI, scale bars 10 μm.

For a more detailed analysis we performed this experiment with LNCs in a size range of 25–100 nm in HEI-OC1 cell lines for 12 h in different dilutions of NPs (Figure 3). FITC was incorporated in the shell, neither payload nor ligand was added. After 12 h incubation in different dilutions of LNC25 (MHD = 25.2 nm), LNC50 (MHD = 53.39 nm), LNC100 (MHD = 102.9 nm), every NP tested showed most uptake in 1:200 dilution (see Figure 3). Further, the LNC25 was internalized most efficiently. The differences in uptake were even statistically highly significant at 200 fold dilution of NPs (LNC25 vs. LNC50 *p* < 0.001; LNC25 vs. LNC100 *p* < 0.001; LNC50 vs. LNC100 *p* = 0.004, Rank-Sum test, *n*small = 12). In 1500 and 15,000 fold dilution no statistical significant difference in uptake could be observed.

**Figure 4:**
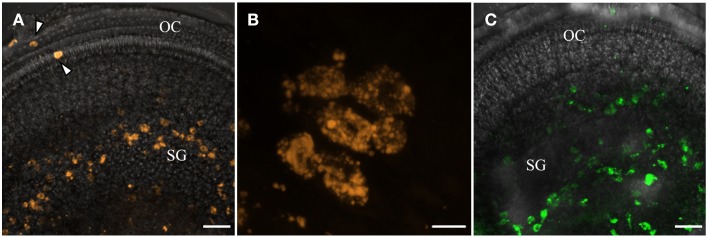
**Internalization of plain bigger QDot NPs shows selectivity for cells in the spiral ganglion (SG) in organotypic cultures with QDot orange (A,B) and QDot green NPs (C)**. Panel **(B)** is a higher magnified view of **(A)** that confirms NPs within the bigger spiral ganglion neuron cytoplasm.

Big QDotNPs (Figure 4) were rather selectively found within cells in the spiral ganglion of organotypic cultures (P3). QDot orange as well as QDot green was located within the cytoplasm of cell somata of putative SGCs and SGNs (Figure 4B) and some cells in the sensory epithelium (arrowhead, Figure 4A), likely macrophage like cells. This result was surprising and suggests an interaction of QDotNPs with neuronal cells at that developmental stage. Since we tested only in an immature model we cannot predict SGNs behavior with mature-by SGC ensheathed-neurons in adult tissue.

### Ligand functionalized nanocarriers

**Figure 5:**
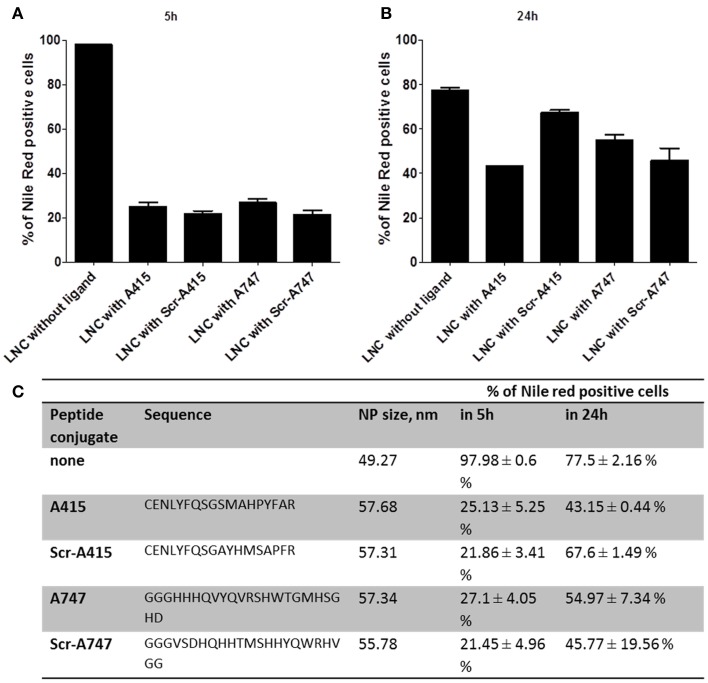
**Flow cytometry results of LNC NP uptake of functionalized TrkB targeting ligands (A415, A747) and scrambled control ligands (Scr-A415, Scr-A747) in comparison with unfunctionalized LNCs after 5 h (A) and 24 h (B) incubation**. Nile Red payload is detected in percentage of positive cells, the table in **(C)** summarized peptide sequence, hydrodynamic NP diameter and quantitative flow cytometry data.

Small peptides with theoretical binding affinity for TrkB receptor (Figure 5C) were tested for specific binding and payload release (Nile red) with LNCs. After 5 days of ATRA treatment SHSY5Y cells were treated with LNC nanoparticles at 50 μg/ml final concentration for 5 or for 24 h, and analyzed by flow cytometry. The unconjugated LNC particles were detectable in almost all cells after 5 h (Figures 5A,C), which decreased to 77% of the cells after 24 h (Figures 5B,C). The peptide conjugated LNC particles were detected in 21–25% of the cells after 5 h and in 43–67% of the cells after 24 h (Figures 5A–C). The targeting or control sequence of the peptide did not have significant effect on the detection of Nile Red in the cells (*p* > 0.05, with One-Way ANOVA). Surface functionalization hampered NP/payload uptake in this cell line, peptide sequence did not show a significant effect. This suggests a peptide sequence independent uptake of LNC particles in SHSY5Y cells. Five hour treatment: Data were not normal distributed using the D'Agostino and Pearson omnibus normality test. Comparison of LNC with A415 with LNC with Scr-A415 using Mann-Whitney U-test: *p* = 0.1242.

Twenty four hour treatment: Data were not normal distributed using the D'Agostino and Pearson omnibus normality test. Comparison of LNC with A415 with LNC with Scr-A415 using Mann-Whitney *U*-test: *p* = 0.100.

**Figure 6:**
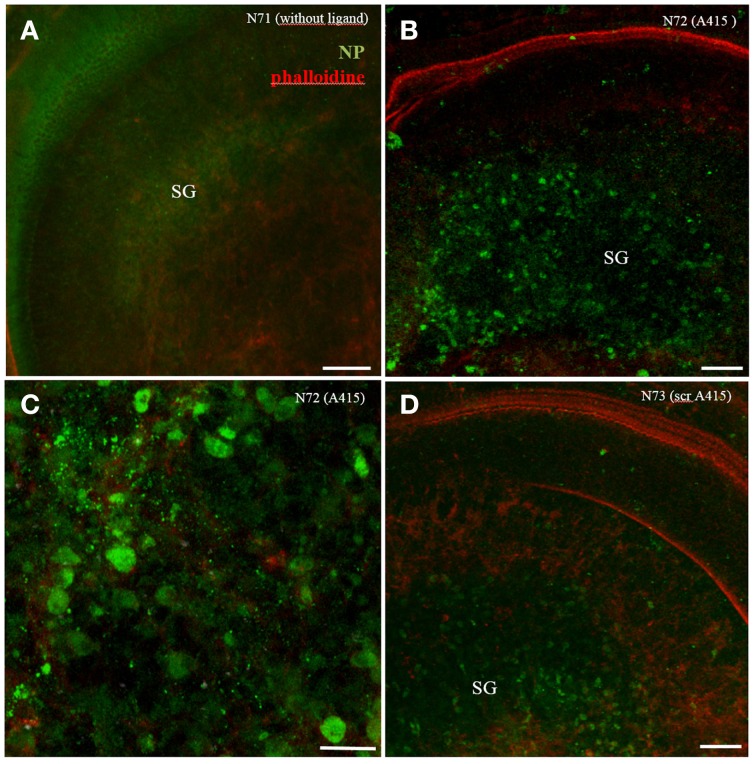
**Organotypic culture with A415 peptide functionalized polymerosom NPs after 24 h of incubation**. While plain LNCs shows even distribution **(A)**, A415-LNC; **(B,C)** and scrambled A415(scr-A415-LNC); **(D)** reveal aggregation in the spiral ganglion (SG) region. Higher magnified view in **(C)** reveals signals mainly within bigger type I neurons. Phalloidine staining (red) indicates stereocilia positions within the sensory epithelium.

A415 ligand conjugated polymerosom NP (FITC in the shell) incubated in cochlear explant cultures for 24 h showed some specifity for neuronal cells in the spiral ganglion (Figures 6B,C), while scrambled peptide showed same specifity but less uptake (Figure 6D, not quantified). Plain polymerosomes showed an even distribution of cellular uptake (Figure 6A). Also in this experiment small peptides change the uptake specifity, here a directional uptake into neurons is visible.

### Extracellular-activated kinase activation with agonistic TrkB antibody

**Figure 7:**
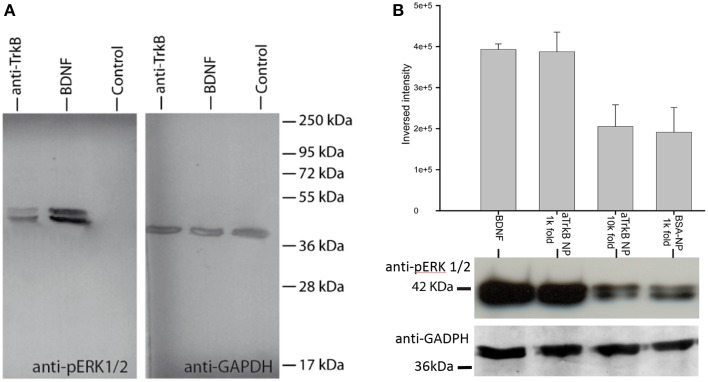
**ERK phosphorylation with anti-TrkB-antibody and anti-TrkB-antibody-functionalized silica NPs (aTrkB-NPs)**. **(A)** Agonistic anti-TrkB antibodies (6B10) without NPs applied to TrkB-overexpressing SH-SY5Y G7 cells induces ERK phosphorylation equivalent comparable to 5.5 nM BDNF application (10 nM BDNF shown). **(B)** Semi-quantitative comparison of ERK-activation by aTrkB-NPs (29D7) within TrkB-overexpressing SH-SY5Y cells compared to BSA-functionalized NPs: aTrkB-NPs in 1000-fold dilution elicited a similar activation of ERK as observed with 5.5 nM BDNF while BSA-functionalized NPs did not. At 10,000 fold dilution there is no significant difference to basal phosphorylation level. Western blot data are shown below.

In order to assess, the capability to activate TrkB-downstream signaling with an agonistic anti-TrkB antibody, the ERK phosphorylation was assessed by western blotting of treated TrkB-overexpressing SH-SY5Y G7 cells. Phosphorylated ERK1/2 from SH-SYS5 G7 cells treated with 10 nM BDNF, 10 nM antiTrkB (6B10) and untreated were compared. After 30 min incubation with antiTrkB, slight ERK1/2 phosphorylation is visible (Figure 7A, Table 1). The phosphorylation is lower than observed with cells treated with 10 nM BDNF but clearly above the basal phosphorylation level.

**Table 1:** **Densitometric analysis SH-SY5Y G7 treated with anti-TrkB (6B10) antibody vs. BDNF presented as ratio over GAPDH expression**.

**Treatment**	**Mean density**	**Mean density**	**Ratio over**
	**GAPDH**	**pERK**	**GAPDH expression**
Anti-TrkB (6B10)	35.002	24.5	0.699960002
BDNF 10 nM	30.477	83.345	2.734685172
Control	39.028	4.14	0.106077688

Silica NP conjugated to 29D7 agonistic antibody (aTrkB NP) also showed effective neurotrophic signaling (Figure 7B). TrkB overexpressing SH-SY5Y G7 cells were treated with 5.5 nM BDNF, 1.9 × E10/ml aTrkB NPs, 1.9 × E9/ml aTrkB NPs and 1.9 × E10/mL BSA-linked NPs. 1.9 × E10/mL. aTrkB NPs were able to induce a phosphorylation response comparable to BDNF. When aTrkB NPs were diluted 10-fold the phosphorylation efficiency dropped to the level of the BSA grafted control particles.

When cells were treated with 5.5 nM BDNF (equals 100 ng.ml^−1^) strong phosphorylation of ERK was induced (Figure 7B). A similarly strong phosphorylation was induced by 1.9 × 10^10^ NPs.ml^−1^ NPs, which carry agonistic TrkB antibodies on their surface immuno-conjugated NP. BSA coated control particle at a concentration of 1.9 × 10^10^ NP.ml^−1^ were not able to induce a similar magnitude of ERK phosphorylation. Also a 10-fold dilution of the aTrkB NPs resulted in a lower ERK phosphorylation (Figure 7B, Table 2).

**Table 2:** **Densitometric analysis SH-SY5Y G7 treated with anti-TrkB (29D7) conjugated silica NP vs. BDNF presented as ratio over GAPDH expression (*N* = 4)**.

**Treatment**	**Mean-pERK to GAPDH**	**SD pERK-related GAPDH**
BDNF 5.5 nM	0.882019403	0.096842924
aTrkB NPs 1 k fold	0.972157927	0.130999313
aTrkB NPs 10 k fold	0.349104961	0.084694112
BSA-NP 1 k fold	0.698961261	0.00317995

### Agonistic TrkB antibody binding tests

**Figure 8:**
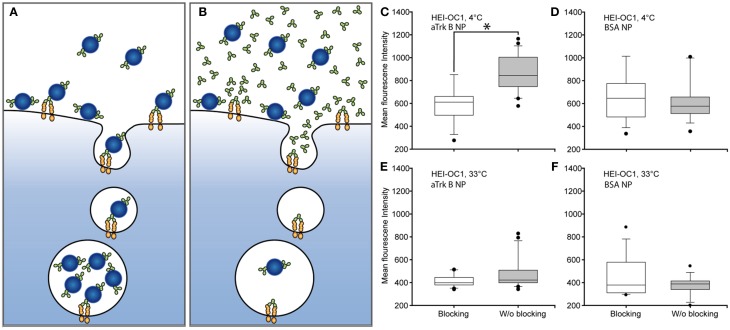
**Assessing the functionality of agonistic anti-TrkB (29D7)-surface grafted silica NPs (aTrkB-NP)**. Principle of competitive binding assays: **(A)** Under binding conditions, aTrkB-NPs are able to specifically und non-specifically bind to the cells. **(B)** Under blocking conditions the excess of free antibody should avoid the specific binding of the aTrkB-NPs leaving only non-specific interactions for binding. In theory a functional antiTrkB surface modification should lead to significantly higher binding and uptake of NPs under binding conditions. Panels **(C–F)** pairwise comparisons of blocking and binding conditions in HEI-OC1 cells. **(C)** HEI-OC1 cells incubated with aTrkB-NPs at 4°C, preventing endocytosis, showed significantly more binding under binding conditions (*p* < 0.05, rank-sum test). **(D)** The same experiment performed with BSA-NP did not show a significant difference (*p* > 0.05, rank sum test). **(E)** When the experiment was performed at 33°C, allowing endocytosis, no difference between binding and blocking conditions was observed (*p* > 0.05, rank sum test). **(F)** The application of BSA-NPs resulted in no difference (*p* > 0.05, rank sum test).

Assessment of the binding capability of our immuno-conjugated NPs was performed in competitive assays (Figures 8A–F). Cells were exposed to aTrkB NPs alone, allowing specific and unspecific binding (Figure 8A) and in a competitive assay with inhibiting specific antibody-mediated binding of the aTrkB NP by an excess of free antibody (Figure 8B). When the experiment was performed at 4°C (Figures 8C,D), preventing endocytosis, under binding conditions, there were significantly more (*p* = 0.001, rank-sum test) NPs attached to the cells than under blocking conditions (Figure 8C). No statistical difference (*p* = 0.450, rank-sum test) was observed when the experiment was performed under identical conditions using BSA-NPs instead of aTrkB NPs (Figure 8D). This suggests an involvement of the antiTrkB-surface modification in the binding of the immuno-conjugated NP. When the experiments were performed at 33°C (Figures 8E,F), allowing endocytosis, there were more aTrkB NPs found to be associated with the cells than under blocking conditions (Figure 8E). However, difference was not significant (*p* = 0.126, rank-sum test). Using control BSA-NP, no significant difference (*p* = 0.142, rank-sum test) between binding and blocking conditions was observed (Figure 8F). These results suggest that under given conditions the surface modification is not playing a significant role for the endocytic uptake of immuno-conjugated NPs.

### Trafficking of antibody conjugated nanoparticles

**Figure 9:**
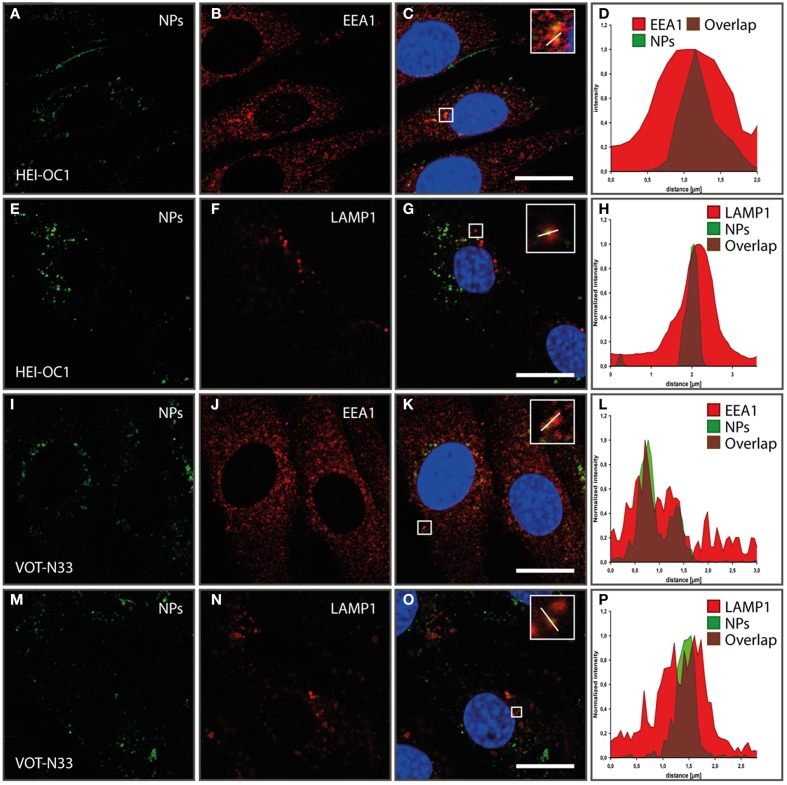
**Internalization and subcellular localization of aTrkB-silica NPs into HEI-OC1 (A–L) cells and VOT-N33 cells (I–P); (A–D)**. In HEI-OC1 cells after 1 h of incubation aTrkB-NPs localize in EEA1 positive endosomes. **(A)** EEA1 staining; **(B)** aTrKB-NPs, **(C)** Overlay; **(D)** luminance profile; scale bars 20 μm. **(E–H)** In HEI-OC1 cells 90 min after start of application aTrkB-NPs were found in LAMP1 positive late endosomes/lysosomes. **(E)** LAMP1 staining; **(F)** aTrkB-NPs, **(G)** Overlay; **(H)** luminance profile; scale bars 20 μm. Panels **(I–L)** in VOT-N33 cells after 1 h of incubation aTrKB-NPs localize in EEA1 positive endosomes. **(I)** EEA1 staining; **(J)** aTrkB-NPs, **(K)** Overlay; **(L)** luminance profile; scale bars 20 μm. Panels **(M–P)** in VOT-N33 cells 90 min after start of application aTrkB-NPs were found in LAMP1 positive late endosomes/lysosomes. **(M)** LAMP1 staining; **(N)** aTrkB-NPs, **(O)** Overlay; **(P)** luminance profile; scale bars 20 μm.

aTrkB NPs were also tested for internalization and subcellular localizations in HEI-OC1 (Figures 9A–H) and VOT-N33 (Figures 9I–P) cells. In both cell lines NPs were found in early endosomes outlined by EEA1 (Figures 9B,J) as well as in LAMP1 (Figures 9F,N) positive late endosomes/lysosomes. After 1 h of incubation aTrKB-NPs localize in EEA1 positive endosomes in both cell lines and after 90 min in LAMP1 positive late endosomes/lysosomes (Figures 9A–H). Like reported previously with unfunctionalized silica NPs (Pritz et al., 2013a) NPs ended in large endosomes with non-overlapping intensity maxima of NP and endosomal marker (Figures 9L,P). We found unfunctionalized silica NPs surrounded by big LAMP-1 positive “rings” to resemble single-membrane compartments with multiple particles at TEM level. Since also in these experiments with aTrKB-NPs few NPs were observed in small vesicles, so clathrin or caveolin mediated endocytosis may be low. The big (>500 nm up to several μm) LAMP-1 positive vesicles resemble macropinosomes like we found with unfunctionalized particles at TEM level previously (Pritz et al., 2013a). This suggests that macropinocytosis may be a dominant uptake mechanism, further no evidence for endosomal escape of these nanocarriers was found.

### Rotary culture of NP-treated inner ears

**Figure 10:**
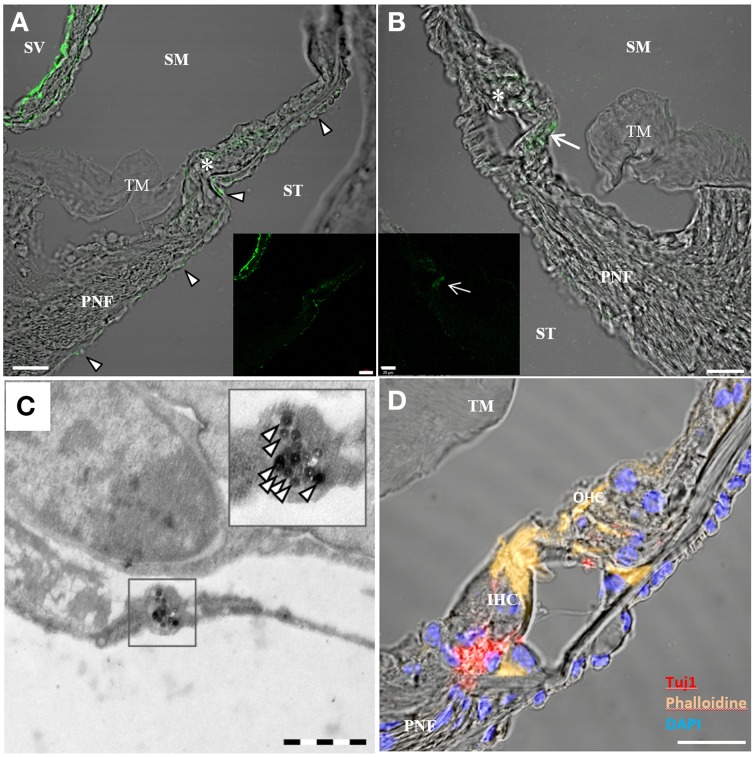
**Confocal imaging on rotary cultures of P25 mouse cochleae treated with aTrkB-silica-NPs**. [**(A,B)** overlay, **(A)** inset green fluorescence, scale bars 20 μm] localized predominantly in the mesothelial covering layer of Reißner's membrane (RM) and tympanic covering layer cells underneath the basilar membrane (arrowheads in **A**). Some staining was observed within the sensory epithelium (asterisks). In **(B)** enhanced uptake was observed within the inner hair cell (arrow). **(C)** Transmission electron microscopy of aTrkB-NPs treated cochlea with NPs found in a tympanic covering layer cell (scale bar 1 μm). **(D)** Confocal projection of a control rotary culture showing the organ of Corti with Tuj1 positive nerve fibers underneath inner (IHC) and outer hair cells (OHC)-scale bar 20 μm. PNF, peripheral nerve fibers; TM, tectorial membrane; SM, scala media; ST, scala tympani; SM, scala media; SV, scala vestibuli.

When whole-organ-cultures were treated with aTrkB NPs for 2 and 8 h, NPs were found to be distributed throughout the cochlea. NPs were observed in the mesothelial/mesemchymal cells lining the perilymphatic fluid spaces. Dense agglomerations of immuno-conjugated NPs were found in the mesothelial layer of Reissner's membrane (Figure 10A) and tympanic covering layer cells underneath the basilar membrane. Infrequently, the aTrkB NPs were observed in the organ of Corti (Figures 10A,B) especially in inner hair cells (Figure 10B) and even less frequently the immuno-conjugated NPs were observed in the spiral ganglion (not shown). In order to overcome the limitations in spatial resolution of light microscopy electron microscopy was performed (Figure 10C). Ultrathin sections were screened for functionalized silica NPs. NPs were found to localize within tympanic covering layer cells of the basiliar membrane (Figure 10C). However, both outer and inner hair cells were not associated with NPs investigated at ultrastructural level. The basal pole of hair cells, where TrkB is exposed by the synaptic terminals, was not found to be associated with NPs. Also the endosomal compartment of hair cells was devoid of aTrkB NPs (not shown). Neither near nerve fibers in the lamina ossea spiralis nor within SGNs were particles detected at ultrastructural level. Based upon these observations, quantitative delivery to TrkB-positive cells such as SGNs and hair cells is very unlikely although some aggregation in inner hair cells is apparent at confocal level that conforms with TrkB expression in adult mice inner ears (Bitsche et al., 2011). Immunostaining of control adult inner ear cultures affirms the presence of an intact and innervated sensory epithelium (Figure 10D).

### Rescuing SGNs with rolipram-loaded LNCs

**Figure 11:**
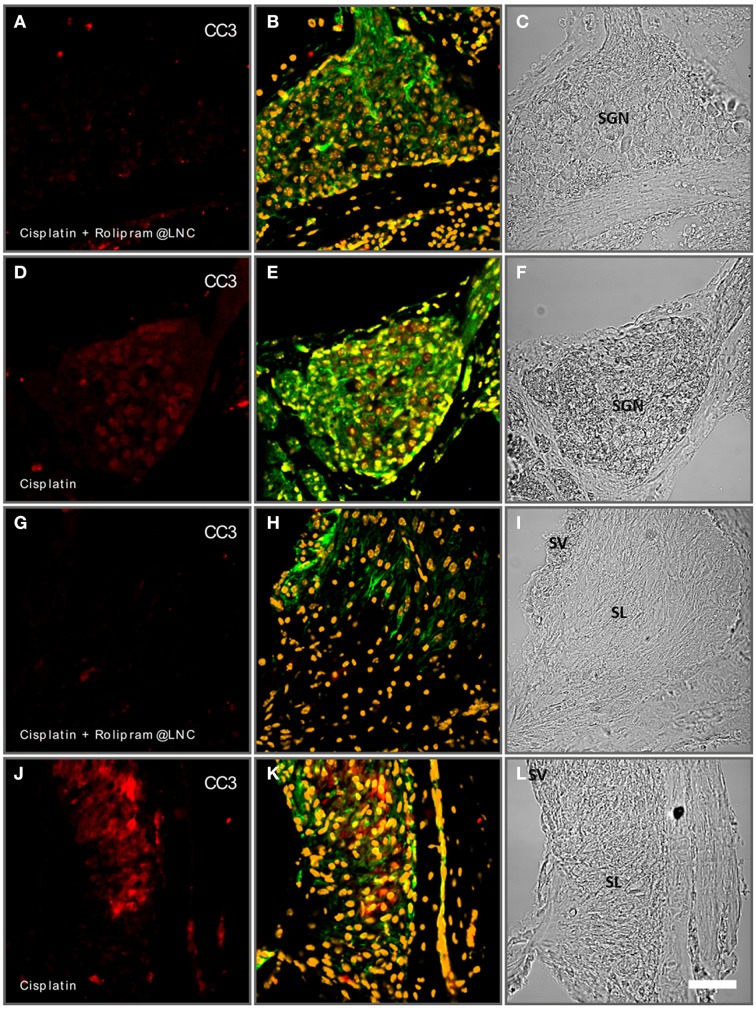
**Rolipram-loaded lipid nanocapsules (LNCs) partially rescue cisplatin-induced apoptosis in postnatal mouse cochlea cultures (P24)**. Spiral ganglion neurons (SGN) treated with cisplatin and rolipram-loaded LNCs exhibit less cleaved caspase 3 (CC3-red staining) immune reactivity **(A)** than controls exclusively treated with cisplatin **(D)**. In the spiral ligament (SL) cisplatin induces increased CC3 reactivity **(J)** while additional treatment with rolipram-loaded LNCs reduced the CC3-reacitivty **(G)**. **(A,D,G,J)** CC3 immunolabeling; **(B,E,H,K)** phalloidine labeling and DAPI staining; **(C,F,I,L)** bright field. Scale bar 50 μm.

Scheper and Colleagues demonstrated (Meyer et al., 2012) that rolipram-loaded LNCs can be used to prevent apoptosis of SGN culture. Here, we employed postnatal rotary culture of whole inner ears to test the potential of rolipram-loaded LNCs in a controllable environment. Rotary culture allows controlling both inducing apoptosis by chemical reagents and the treatment by NPs in controlled concentrations. In our model, we induced apoptosis by the application of 50 μM cisplatin (Figure 11). In cisplatin-treated cochleae, we observed a rise in CC3 immunoreactivity after 24 h of treatment (Figure 11D). However, when rolipram-loaded LNCs diluted 1000-fold applied together with cisplatin, the CC3 immunoreactivity was significantly reduced (Figure 11A). Analogous to the SGN similar effects were observed in the spiral ligament, which appears to be susceptible to apoptosis in the, cochlea culture. In this part of the cochlea, CC3 levels could also be rescued by the application of rolipram-loaded LNCs (Figures 11G,J). Controls are summarized in an Supplementary Figure 1. Together, this demonstrates that mouse rotary cultures of the cochlea is an efficient and sensitive test bed for the pharmacotherapy of the NPs. The rotary culture allows for exact control of concentration of reagents. It excludes the detrimental influence of animal behavior and is perfectly compatible with imaging-based analysis and biochemical interrogation.

## Discussion

In the present study we demonstrated some principle application of nanocarriers for use of drug delivery to the inner ear. NP size influences uptake kinetics and may reflect different pathways into the cells. Mironava et al. reported that 45 nm Gold NPs penetrate dermal fibroblasts via clathrin-mediated endocytosis, while smaller 13 nm Gold NPs enter mostly via phagocytosis (Mironava et al., 2010). Mesoporus silica NP showed maximum uptake at a NP size of 50 nm; 30 and 110 nm showed significantly less uptake by HeLa cells (Lu et al., 2009). A NP core size of 30–50 nm seem to be an optimal size for active uptake (Shang et al., 2014) for most cells. Small spherical NPs have less interaction with the cell membrane, so small NP have to aggregate at the cell surface to trigger internalization (Jiang et al., 2010). Although we controlled NP composition and charge and used serum free media supplements, the NP corona will have been altered by some of these supplements and proteins secreted by cells and inner ear explants. Therefore, we can only speculate on uptake mechanisms varying with NP size. For unfunctionalized 50 nm silica particles we found macropinocytosis to be most relevant privileged by *in vitro* formation of NP agglomerates (Pritz et al., 2013a) and confocal imaging on the TrkB functionalized here show similar results. NP aggregation under *in vitro* conditions is poorly understood. Material properties such as particle size, morphology, and crystallinity are important parameters affecting nanoparticle colloidale stability (Gambinossi et al., 2014). The impact of aggregation cannot not easily predicted and needs to be evaluated NP by NP. Unfunctionalized LNCs highly penetrate cells and tissue of the inner ear and are able to release their cargo such as rolipram. LNCs are chemically stable and ideal to encapsulate hydrophobic cargos at high encapsulation rates. They displayed clear size dependency of internalization and did not show signs of toxicity compared to control experiments. In cochlear explants an enhanced uptake was identified for immature SGCs. Like in dorsal root ganglia there are cells to migrate from the neural crest and have the potential to generate glia and more than 20 different neuronal cell types, many of these cells undergo apoptosis (Raible and Ungos, 2006). The cells responsible for dead dorsal root ganglia neurons removal are satellite glial cell (SGC) precursors, rather than macrophages (Wu et al., 2009). These characteristics might be valid also for SGC in the immature cochlea and may explain enhanced uptake. Polymerosomes confirms that size is an important factor for kinetics of tissue penetration. On the big end of our NP portfolio QDot NPs demonstrated that also particles of several hundreds of nm show capability for uptake in SGCs and SGNs. Although we clearly found these particles also in the bipolar neurons uptake by immature SGCs and transcytosis to SGN may occur here, especially with those neurons that are already completely covered by their satellite sheath. Further, analysis might be valuable to study this possible pathway of NP transport.

As NPs are functionalized with small peptides internalization characteristics changes considerably. In our *in vitro* system using SHSY5Y cells the peptide functionalized LNCs showed worse uptake than the non-functionalized ones. In fact, this observation is in agreement with a previous report (Hirsjarvi et al., 2013). LNCs are subjected to surface modifications, including charged molecules (Perrier et al., 2010). Attaching for instance polysaccharides to the surface of LNCs offers the possibility of modifying the physicochemical and biological properties of the core particles. Surface modification of LNCs by post-insertion of the positively charged amphiphilic (Perrier et al., 2010) lipochitosan made the LNC surface more rigid, but the neutral, higher molecular weighted lipodextran had no effect on the surface elasticity (Hirsjarvi et al., 2013). Although, these surface modified LNCs were better captured by the mononuclear phagocyte system, the neutral and charged molecules did not influence the *in vivo* biodistribution properties of LNCs in mice (Hirsjarvi et al., 2013). In addition, neuronal uptake mechanisms may not be identical with the mononuclear phagocyte system. With polymerosomes we found similar results in organotypic cultures. Here unfunctionalized particles showed an even uptake throughout the tissue while pattern differed with functionalized NPs. Here again a preference for the region of the spiral ganglion was found and appeared to be enriched with the targeting ligand. Further experiments investigating the kinetics of uptake in certain cell types would be necessary to prove specifity. However, adding moieties that can be used to target NPs into different cell types with high affinity is not an easy task. Surface modification of NPs often need to be introduced for providing functional groups that ca be conjugated with the ligand thereby changing the characteristics in and of themselves. To ensure ligand directed coupling with correct orientation and desired surface density may be a problem especially for very small particles (Friedman et al., 2013).

Antibody grafted NPs also face major challenges such as high specifity and affinity for target structures on cell surfaces. Further linker and nanocarrier material must not lower specific binding. Initial tests whether our antibody surface modification is functional and contributes both to enhanced binding and internalization in TrkB positive cells showed that the surface modification contributed to binding only. TrkB positive HEI-OC1 cells were exposed to functionalized NPs or to functionalized NPs and an excess of TrkB antibody inhibiting antibody-mediated binding of the immuno-NPs. When the experiment was performed at 4°C, inhibiting endocytosis, significantly more NPs were found on cells treated with only immuno-NPs than on cells treated with both free antibody and functionalized NPs. However when, the experiment was carried out at 33°C the significant difference was lost. This suggests that the binding of the functionalized NP is significantly determined by the specific antibody-TrkB interaction while the contribution of the antibody to the endocytosis of the membrane associated NP is negligible. This interpretation might be counterintuitive but it fits to the model of Decuzzi and Ferrari (2007). According to their model, for a NP the contribution of non-specific interactions such as electrostatic force to the early stages of endocytosis is as important as the specific interactions which are exerted by ligand surface modifications. The herein used 50 nm silica NP had an average of 3–5 antibodies grafted to the surface; the larger part of the NP surface was accessible to unspecific membrane-to-NP interactions. As a consequence, attractive unspecific interactions between NP and membrane might have been considerably larger than the specific interactions, thus governing the membrane wrapping process. Since the formation of the membrane invagination is the step determining the velocity in the endocytic process, the avidity of the immuno-conjugated NP-TrkB interaction might be insufficient to contribute with significant, specific interactions between NP and cell. It might appear that under given conditions the unspecific binding of NP to the cell is more significant for the endocytic uptake than the antibody-mediated binding. Taken together, this suggests for the herein used NPs the surface-grafting of TrkB antibodies was insufficient to significantly contribute to endocytosis of the NP.

A further explanation for the obtained results might be provided by macropinocytosis as source for “non-specific” internalization of the immuno-NPs. Since the aTrkB NPs were composed of the same matrix material as the NPs used previously (Pritz et al., 2013a), macropinocytosis might also have contributed substantially to the internalization of the aTrkB NPs as reported in the this study. In case of macropinocytosis, large amounts of extracellular liquid are internalized into the cell. Therefore, the internalization of NPs by macropinocytosis does not necessarily require membrane binding of the NPs. A high rate of non-specific ground-level internalization by macropinocytosis would explain the lacking difference between the binding and the blocking set-up. A factor which would lead to an increase in macropinocytosis-dependent internalization is NP clustering (Canton and Battaglia, 2012). As the binding and internalization experiments were conducted under serum-free conditions, only the “blocking” set-up with its 63-fold antibody excess provided enough soluble protein to cause protein-mediated NP-aggregation (Monopoli et al., 2011). An antibody-induced NP agglomeration could have led to an artificially higher internalization rate under “blocking” conditions. Moreover, also an excess of the aTrkB NPs itself might have been responsible for an increased non-specific binding of the immuno-NPs. The higher the concentration of the NPs, the higher the possibility for a non-specific pinocytic internalization by macropinocytosis. These considerations suggest that also a better control of the discussed experimental parameters as well as the colloidal stability is necessary to assess the true contribution of the antibody surface modification to the endocytosis of the immuno-NPs.

The insufficient contribution of NPs to internalization and tissue penetration suggest that an optimization of the nanocarrier system to selectively bind and activate TrkB with specific uptake in TrkB positive cells is necessary. Since a mismatch between specific and unspecific NP-to-membrane interactions is a likely source of error, an increase in specific interaction strength and a decrease in non-specific interaction would be beneficial. This can be achieved by increasing the number of antibody molecules per NP. Silica NPs expose amino residues for surface functionalization. The remaining amino residues might be used for surface PEGylation. This would efficiently decrease non-specific interactions with the biological environment. For example, such a polymer brush is thought to minimize NP-protein-interactions (Vonarbourg et al., 2006a,b; Monopoli et al., 2011). The polymer coating could prevent non-specific interactions with the membrane/membrane proteins. In general a change of the particle type would also be an approach to solve these problems. LNCs would offer optimal membrane interaction properties. One the one hand, they minimize unwanted interactions by a PEG brush. On the other hand they exhibit a 10-fold higher avidity caused by a higher number of antibody molecules on the particle surface (Beduneau et al., 2007, 2008). However, we showed that there were enough aTrk-NPs or aggregates bound to TrkB positive cells present to activate neurotrophic signaling. This might partially compensate the slowing down and impairment of endocytosis as observed with unfunctionalized silica NPs (Pritz et al., 2013a). Immuno-conjugated NPs showed similar trafficking pattern at confocal level that may pose a threat to important functions in the survival signaling in SGNs. Thus, (over-) load of silica NPs for cell types involved in sensorineural survival signaling needs to be minimized and a strategy for endosomal escape has to be applied for a use as drug carriers. In conclusion the surface chemistry of NPs aimed for TrkB-positive SGNs has still to be optimized and tested to guarantee a functional and specific antibody-based DD system customized for the SGNs.

Concerning the application of immuno-NPs to the whole-organ culture of cochleae, consistent results from TEM and confocal imaging show that the tissue permeation of the immuno-NPs was insufficient to reach TrkB positive cells in higher amounts. Antibody contribution to internalization of the immuno-NP-TrkB-complex is low as shown with our competitive blocking assay. TrkB is expressed in SGN in adult human (Liu et al., 2011) and in inner hair cells and neurons in young adult mice (Bitsche et al., 2011). Although some aggregation to inner hair cells was found our observations suggest that the permeation of the aTrkB NPs to those sites was not quantitative. The lack of tissue permeation might be caused by the specific physicochemical parameters of the silica NP. Since it was documented for *in vivo* experiments with other particles like LNCs (Zou et al., 2008), polymerosomes and liposomes (Buckiova et al., 2012) to locate within the cells of the organ of Corti, it might be necessary to switch to one of those carrier types. As a consequence, a fundamental change in the physicochemical properties of the carrier or a change to another type of nanocarrier is required to reach these TrkB-positive structures in the cochlea. Another problem for DD via the RWM into the ear presents the high phagocytic activity of the mesothelial covering layer cells lining the perilymphatic spaces. These cells form a loose layer. Like in other body cavities this cell type is able to phagocyte even bacteria and has function for antigen presentation (Visser et al., 1996). Strategies to selectively prevent this phagocytosis or better promote transcytosis toward endolymphatic compartments may be necessary. Analysis of fluid spaces in the human cochlea suggests rather easy accessibility for from scala tympani to SGNs (Rask-Andersen et al., 2006).

DD with targeting ligand NPs for the inner ear remain complex nevertheless the inner ear may be a good model organ for this approach with the possibility for local drug delivery to an organ isolated by a blood-labyrinth-barrier. Schuknecht was one of the first who experimented with intratympanic drug administration in the treatment of severe vertigo (Schuknecht, 1956). He reported success in Meniere symptomology with the intratympanic streptomycin injection but resulted also in profound hearing loss. However, the efficacy of his approach vs. systemic drug administration has been confirmed for various clinical indications. The major site of absorption of drugs is the RWM, the concentration of the drug in the inner ear depends greatly on the exposure time and drug concentration. To overcome rapid clearance by ciliated epithelia in the middle ear different hydrogels were used to immobilize a drug reservoir (Paulson et al., 2008). They tested chitosan-glycerophosphate hydrogels containing dexamethasone or gentamicin in the murine cochlea on the round window. We also previously reported permeation and distribution of paramagnetic particles loaded in an intelligent gel becoming solid at body temperature (Thaler et al., 2011). A cochlear implant is an artificial hearing device that can replace damaged hair cells in the cochlea. To minimize trauma during electrode insertion and reduce postsurgical inflammation reactions and fibrosis becomes more and more important especially in context with electro-acoustic stimulation of the cochlear nerve. Drug eluting electrodes are currently developed also using hydrogels (Hutten et al., 2014) or polymeric coatings (Ceschi et al., 2014). These carrier materials offer also great possibilities for nanocarriers and are already in use with human applications (Nakagawa et al., 2014).

Kikkawa et al. (2014) tested insulin like growth factor or heptatocyte growth factor in gelatine hydrogel coated electrodes. Their findings provide the first evidence that a hydrogel coated, growth factor-releasing electrode could attenuate insertion trauma and promote recovery from it. A combination that might be a DD strategy also for other inner ear damages.

Our experiments with rolipram loaded LNCs proved drug release by a nanocarrier. The drug delivery mechanism of LNCs is believed to be dependent on endocytosis and to involve endosomal escape (Paillard et al., 2010). For lipophilic cargo a slow and constant release from the LNC and a subsequent diffusion to the acceptor compartment has been demonstrated to accomplish subcellular DD with our HEI-OC1 cell line without the involvement of endocytosis (Bastiat et al., 2013). In contrast, amphiphilic cargo is confined to the LNC lumen. While amphiphilic cargo is confined in the surfactant shell of the LNC, lipophilic molecules are localizing in the lipophilic center of the LNCs, allowing diffusion of the cargo. Our rolipram loaded LNC NP in rotary cultures of adult inner ears demonstrate the bioefficacy of this drug release system to prevent apoptosis caused by cisplatin in the adult murine inner ear. Previous experiments could prove that incorporation of rolipram in LNCs increased the survival of SGNs significantly in *in vitro* cultures of immature neurons (Meyer et al., 2012). Without the use of any nanocarriers, concentration level of rolipram appeared to be very crucial as demonstrated in Kranz et al. (2014). Our rotary culture system proved as controllable system for adult inner ear tissue and could demonstrate this effect for the first time in adult inner ear tissue. LNCs may act as an appropriate system to deliver lipophilic drugs in low doses with a linear release characteristics suitable for inner ear application and do not feature initial burst release (Lamprecht et al., 2002). Thus, LNCs are one of the few carrier systems, which were able to demonstrate actual DD efficiency in the cochlea so far.

### Conflict of interest statement

The authors declare that the research was conducted in the absence of any commercial or financial relationships that could be construed as a potential conflict of interest.

## Supplementary material

Supplementary Figure 1 **Control preparations for Rolipram-loaded lipid nanocapsules (P24, 48 hours incubation)**. Untreated spiral ganglion neurons (SGN) stained for cleaved caspase 3 (CC3-red staining) immune reactivity shows only few staining **(A)** as well as in the spiral ligament (SL) **(D)**. Treatment with rolipram (14 μM) shows no effect on CC3-reactivity **(G,J)**. **(A,D,G,J)** CC3 immunolabelling; **(B,E,H,K)** phalloidine labelling and DAPI staining; **(C,F,I,L)** bright field. Scale bar 50 μm.
