# Supplementary figures and images for: Nanoparticle mediated drug delivery of rolipram to tyrosine kinase B positive cells in the inner ear with targeting peptides and agonistic antibodies
Source: Front Aging Neurosci. 2015 May 19;7:71. doi: 10.3389/fnagi.2015.00071 (PMC4436893; doi:10.3389/fnagi.2015.00071)

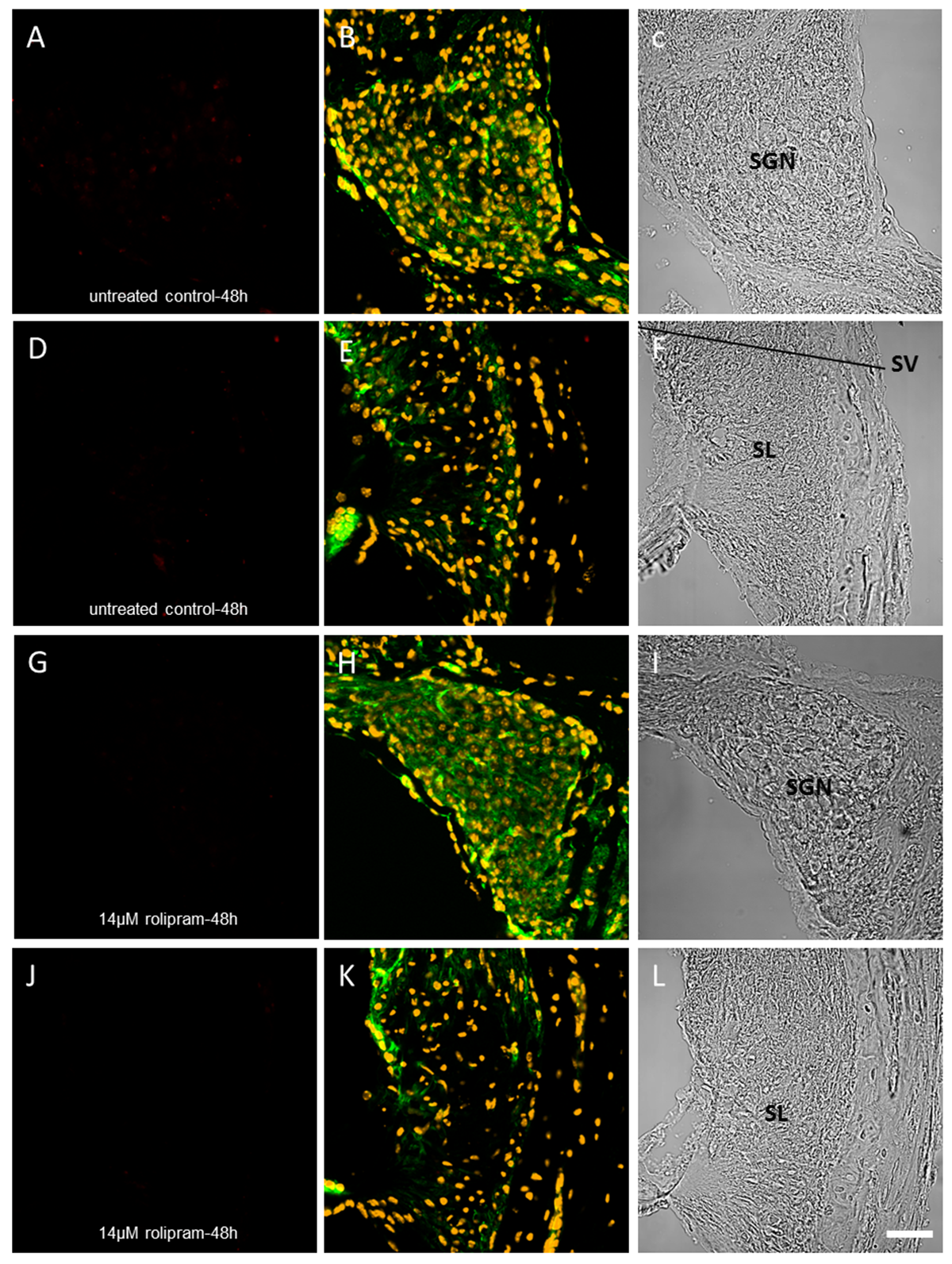

Supplement: Supplementary Figure 1 — Control preparations for Rolipram-loaded lipid nanocapsules (P24, 48 hours incubation). Untreated spiral ganglion neurons (SGN) stained for cleaved caspase 3 (CC3-red staining) immune reactivity shows only few staining (A) as well as in the spiral ligament (SL) (D). Treatment with rolipram (14 μM) shows no effect on CC3-reactivity (G,J). (A,D,G,J) CC3 immunolabelling; (B,E,H,K) phalloidine labelling and DAPI staining; (C,F,I,L) bright field. Scale bar 50 μm. [file Image1.TIF]
